# The Ess/Type VII secretion system of *Staphylococcus aureus* shows unexpected genetic diversity

**DOI:** 10.1186/s12864-016-2426-7

**Published:** 2016-03-11

**Authors:** Ben Warne, Catriona P. Harkins, Simon R. Harris, Alexandra Vatsiou, Nicola Stanley-Wall, Julian Parkhill, Sharon J. Peacock, Tracy Palmer, Matthew T. G. Holden

**Affiliations:** The Wellcome Trust Sanger Institute, Wellcome Trust Genome Campus, Hinxton, Cambridge, CB10 15A UK; University of Cambridge, Addenbrooke’s Hospital, Cambridge, CB2 0QQ UK; Division of Molecular Microbiology, College of Life Sciences, University of Dundee, Dundee, DD1 5EH UK; School of Medicine, University of St Andrews, St Andrews, KY16 9TF UK

**Keywords:** *Staphylococcus aureus*, Secretion, Type VII

## Abstract

**Background:**

Type VII protein secretion (T7SS) is a specialised system for excreting extracellular proteins across bacterial cell membranes and has been associated with virulence in *Staphylococcus aureus*. The genetic diversity of the *ess* locus, which encodes the T7SS, and the functions of proteins encoded within it are poorly understood.

**Results:**

We used whole genome sequence data from 153 isolates representative of the diversity of the species to investigate the genetic variability of T7SS across *S. aureus*. The *ess* loci were found to comprise of four distinct modules based on gene content and relative conservation. Modules 1 and 4, comprising of the 5’ and 3’ modules of the *ess* locus, contained the most conserved clusters of genes across the species. Module 1 contained genes encoding the secreted protein EsxA, and the EsaAB and EssAB components of the T7SS machinery, and Module 4 contained two functionally uncharacterized conserved membrane proteins. Across the species four variants of Module 2 were identified containing the *essC* gene, each of which was associated with a specific group of downstream genes. The most diverse module of the *ess* locus was Module 3 comprising a highly variable arrangement of hypothetical proteins. RNA-Seq was performed on representatives of the four Module 2 variants and demonstrated strain-specific differences in the levels of transcription in the conserved Module 1 components and transcriptional linkage Module 2, and provided evidence of the expression of genes the variable regions of the *ess* loci.

**Conclusions:**

The *ess* locus of *S. aureus* exhibits modularity and organisational variation across the species and transcriptional variation. *In silico* analysis of *ess* loci encoded hypothetical proteins identified potential novel secreted substrates for the T7SS. The considerable variety in operon arrangement between otherwise closely related isolates provides strong evidence for recombination at this locus. Comparison of these recombination regions with each other, and with the genomes of other Staphylococcal species, failed to identify evidence of intra- and inter-species recombination, however the analysis identified a novel T7SS in another pathogenic staphylococci, *Staphylococcus lugdunensis*.

**Electronic supplementary material:**

The online version of this article (doi:10.1186/s12864-016-2426-7) contains supplementary material, which is available to authorized users.

## Background

The secretion of virulence factors is an essential process for bacterial pathogenesis, and bacteria have evolved numerous systems through which proteins can be secreted into the environment or injected into host cells [1]. The Type I through Type VI secretion systems are found in Gram negative bacteria and mediate the transport of protein substrates across the two membranes of the cell envelope, in either a one-step or two-step mechanism. The Type VII secretion system (T7SS, variously known as the ESX-1 or ESAT-6 secretion system), by contrast, has not been functionally described in Gram negative bacteria, but is found in the Gram positive Actinobacteria and Firmicutes [2–4]. It was initially identified in pathogenic mycobacteria, with *Mycobacterium tuberculosis* secreting two T-cell antigens (termed ESAT-6/EsxA and CFP-10/EsxB) via the T7SS pathway. There is significant evidence that this system is an important virulence factor in mycobacteria. Genes encoding ESAT-6 and CFP-10 form part of the region of difference 1 (RD1), a cluster of genes that is deleted from the genome of the *M. bovis* BCG vaccine strains [5]. This deletion has been linked to the reduced virulence of the BCG strain [6], and evidence in murine models has demonstrated the importance of the ESX-1 system in enabling bacterial translocation from the phagolysosome into the cytosol, a key step in mycobacterial virulence [7].

The mycobacterial T7SS comprises a number of membrane proteins that form a large 1.5 MDa complex [7]. Central to the complex is EccC, a transmembrane protein which has three globular domains of the SpoIIIE-FtsK-like ATPase family [2]. Structural analysis of EccC has shown that the most C-terminal ATPase domain interacts with the signal sequence of the secretion substrate EsxB, which promotes oligomerisation of EccC and activates its ATPase activity [8]. EsxB and the related substrate protein EsxA are founding members of the WXG100 protein superfamily that are characterised as small helical hairpin proteins with a centrally positioned Trp-Xaa-Gly (WXG) motif [9]. Other substrates of the mycobacterial T7SS are the Pro-Glu (PE) and Pro-Pro-Glu (PPE) proteins that are larger than EsxA/EsxB but show a similar helical hairpin arrangement. It is likely that WXG100 substrates are exported as folded dimers [10].

Homologs of the mycobacterial T7SS components EsxA, EsxB and EccC are also encoded by some firmicutes and secretion of EsxA and/or EsxB have been demonstrated in *Bacillus subtilis* [10, 11], *Bacillus anthracis* [12] and *Staphylococcus aureus* [13, 14]. However several of the essential mycobacterial T7SS components are not found among the firmicute T7SS, with only the EccC-like ATPase and one or both of EsxA and EsxB being common across the phyla [3]. This has led to the Firmicutes’ systems being designated Type VIIb to distinguish them from the well-characterised mycobacterial secretion system [2].

*S. aureus* is a human commensal bacterium and an opportunistic pathogen that can cause a broad range of clinical manifestations in humans, including the majority of skin and soft tissue infections and a substantial proportion of invasive infections such as endocarditis and osteomyelitis [15–17]. The genes encoding the T7SS are found at the *ess* (ESAT-6-like secretion system) locus and are highly up-regulated during long-term persistence in the cystic fibrosis airway, consistent with a role in persistent infection [18]. Collectively, studies with *S. aureus* strains Newman, USA300 and RN6390 have shown that the *ess* locus codes for two secreted WXG100 family proteins (EsxA and EsxB) and two secreted proteins lacking the WXG100 motif (EsxC and EsxD). Three *ess*-encoded transmembrane proteins, EssA, EssB and EssC, are reportedly essential for protein secretion, but a potential function has been assigned to only one of these, the EccC-like ATPase EssC. The ubiquitin-like protein EsaB has been proposed to regulate Ess activity at either the post-transcriptional or post-translational level. Finally, the membrane protein EssD also encoded at this locus, has a non-essential role in protein secretion [13, 19–21].

*S. aureus* is a clonal bacterial species and is dominated by a number of successful lineages [22]. Between members of different *S. aureus* lineages there is variation in the extensive arsenal of immune evasion and virulence factors that modulate the host cell interaction under complex genetic regulation [23]. The *ess* locus has previously been described in a small number of *S. aureus* strains from multi-locus sequence type (MLST) clonal complex (CC) 8 (strains Newman [13, 14, 19, 21], USA300 [14, 19, 20], RN6390 [14], COL [14], SA113 [14]), CC5 (Mu50 [19, 24], N315 [3]) and CC1 (MW2 [19]). Comparative genomic analysis identified the *ess* locus as one of the relative few core variable regions in the *S. aureus* genome. However, a study of CC30 strains derived from the airways of a cystic fibrosis patient chronically infected with *S. aureus* found no transcription of a number of T7SS genes, including *esxB*, *esxC* and *essD*; subsequent sequencing of these strains found that these genes were missing from the isolates’ genomes [18]. Sequence similarity searches of published *S. aureus* genomes identified that in 12 strains these genes were missing, illustrating genetic diversity of the *ess* operon across the species.

In this study we describe the genetic diversity and organisational variation within the *ess* locus, using sequence data from a broad range of *S. aureus* strains that captures diversity across the species. Conducting bioinformatics analysis we have explored the diversity of this secretion system in *S. aureus* and postulate potential functions of additional hypothetical proteins encoded at the *ess* locus that are likely to contribute to the T7SS.

## Results

### Genes comprising the *ess* locus can be described in four distinct modules

**Fig. 1:**
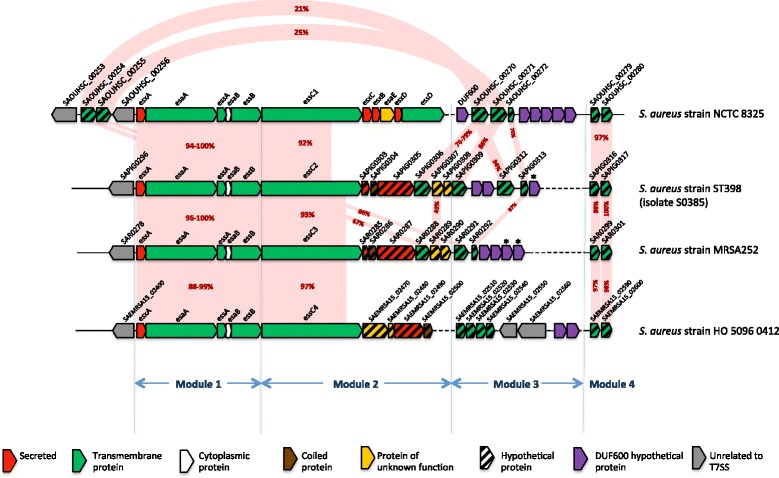
The modular structure of the T7SS loci of *S. aureus*. Schematic representation of the *ess* operon in four reference genomes strains of *S. aureus*, demonstrating the four modules that describe variation in the operon and the four major variants of *essC* and its immediate downstream gene cluster: *Module 1.* A conserved cluster of five genes (*esxA* to *essB*) at the 5’ end of the operon that encode one secreted protein (EsxA) and other components of the T7SS machinery; *Module 2.* A variable region that includes the *essC* gene, of which there are four variants, each associated with a distinct cluster of four to six downstream genes that are predicted to encode a number of secreted and transmembrane proteins; *Module 3.* A highly variable region which encodes a number of predicted transmembrane proteins and proteins possessing the domain of unknown function (DUF600); and *Module 4.* A 3’ conserved region, encoding two highly conserved hypothetical proteins of unknown function. Light pink shading demonstrates regions that are conserved between isolates, with the degree of amino acid identity between isolates displayed in red text. An asterisk above the gene indicates that it is present as a pseudogene

Comparative genomic analysis demonstrated that the *ess* locus had a complex gene arrangement, which varied both between and within clonal complexes. To facilitate description of this variation we divided the region into four distinct modules, a schematic of which is shown in Fig. 1. The rationale for this was based on the observation that genes in Modules 1 and 4 were present in most isolates and were largely conserved; Module 2 comprised a gene cluster that occurred as four distinct variants; and Module 3 possessed the greatest variation, including several genes encoding hypothetical proteins that varied even between otherwise closely-related isolates.

### Module 1: the *esxA* gene and the four downstream genes are conserved in all clonal complexes

Module 1 contained the first five genes of the *ess* locus (*esxA*, *esaA*, *essA*, *esaB* and *essB*), homologs of which were present in all but one of the sequences analysed. The gene arrangement of Module 1 was as described previously [3, 13, 20], with little variation in the predicted amino acid sequence in the study isolates as presented in Fig. 1. The highly conserved nature of these genes is consistent with functional analysis showing that they encode essential components of the secretion machinery [20, 21]. A single isolate that lacked *esxA*, *esaA*, *essA*, *esaB* and *essB* (ASASM86) contained a genomic deletion of 9166 bp that resulted in the loss of these genes.

### Module 2: the 5’ portion of *essC* is conserved, but there are four variants of the *essC* 3’ region

The *essC* gene encodes a membrane-bound protein with three C-terminal ATPase domains and is a key component of all T7SS. Homologs of this gene were identified in all of the *S. aureus* isolates investigated. The 5’ region of this protein coding sequence (CDS) (approximately 3220 bp of *essC*) was conserved among all isolates, however the 3’ region (approximately 1230 bp of *essC*) and downstream CDS fell into four distinct variants. We have termed these variants *essC1-4* (Fig. 1). The *essC1* variant had the highest frequency (90/153 isolates) and was found in strains belonging to CCs 1, 5, 7, 8, 9, 25, 41, 51 and 88. These included the reference strains Newman [25], USA300 [26], COL [27], Mu50 [28], N315 [28] and MW2 [29], which have previously been used to describe the biology and function of the EssC protein [3, 14, 19, 20, 24]. The next most abundant variant was *essC3* (41/153 isolates), which was found in CC30 and ST239, and included the TW20 [30] and MRSA252 [31] reference isolates. By contrast, the *essC4* variant was only identified in isolates belonging to CC22 (including the EMRSA-15 reference isolate HO 5096 0412) [32], and *essC2* was identified in CC15 and ST398 (including the ST398 reference genome, isolate S0385) [33]. A summary of the *essC* variants associated with each isolate is included in Additional file 1: Tables S1 and S2. For illustrative purposes we have chosen the *ess* loci of NCTC 8325, S0385, MRSA252 and HO 5096 0412 reference genomes strains as representative of the four *essC* variants, *essC1*, *essC2*, *essC3* and *essC4* respectively.

**Fig. 2:**
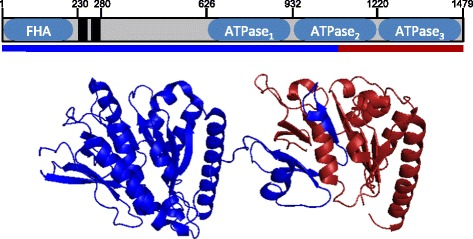
Domain architecture of EssC protein. Sequence variable region of EssC shown in red on the X-ray structure of the C-terminal ATPase domains of *G. thermodentrificans* EssC. The blue and red line underneath the linear representation of EssC marks the extent of the conserved and variable regions identified in the comparison between the *essC1* and *essC2* illustrated in Fig. 1

It was notable that the sequence divergence associated with this module at the 5’ end occurred within the *essC* gene. The X-ray structure of the C-terminal 550 amino acids of EssC from *Geobacillus thermodenitrificans*, a thermophilic member of the firmicutes phylum has been reported [8] and encompasses two of the three ATPase domains. We mapped the variability observed in the EssC sequences onto the structure of *G. thermodentrificans* EssC. It is clear from Fig. 2 that the sequence variability largely encompasses the final ATPase domain. Since this C-terminal domain in the actinobacterial EccC is involved in substrate recognition [8, 34], this raises the possibility that the four EssC variants of *S. aureus* recognise different repertoires of secreted proteins due to the C-terminal domain variation.

The five genes immediately downstream of *essC1* have been previously demonstrated to be co-transcribed with the genes encoding the core secretion components [14]. Three of the five genes, *esxC*, *esxB* and *esxD* encode secreted T7SS substrates [13, 19, 20] whilst *essD* encodes a transmembrane protein and *esaE* a predicted soluble protein of unknown function [13, 19–21]. Although every isolate with the *essC1* variant in this study had the same arrangement of these five genes, by contrast none of the isolates with the other three *essC* variants (*essC2*, *essC3* and *essC4*) possessed any of these genes. Instead, each *essC* variant (Fig. 1) was associated with a unique combination of downstream genes that constituted Module 2. Since it is known for strains with *essC1* sequences that Module 2 genes encode secreted substrates and accessory proteins, these findings strongly suggest that the EssC sequence variants are associated with a distinct repertoire of secreted proteins and accessory factors.

### Prediction of function for Module 2 genes

Each of the *essC* module variants contained at least one gene encoding a protein with a WXG domain that are proposed to be secreted by T7SS (Fig. 1; *esxB*, *SAR0287*, *SAEMRSA15_2490* and *SAPIG0305*). Consequently, each variant Ess system has at least two WXG family proteins: an EsxA protein (found in Module 1) that is highly sequence-conserved regardless of EssC subtype and a Module 2 protein, conserved with the same EssC subtype but very poorly conserved across strains with different EssC subtypes. Additional proteins sharing sequence similarity with the WXG100 family of secreted proteins were identified in the *essC2* and *essC3* variants (*SAPIG0303* and *SAR0285* respectively; Fig. 1). Although these two proteins lack the WXG motif associated with other secreted proteins in the T7SS, Interpro identified them as potential T7SS effectors in the SACOL2603 family of proteins, which are similar in length and share some sequence similarities with the WXG100 protein family.

Although functions for remaining CDSs in Module 2 could not be predicted, the conservation of protein motifs implies conserved function in this cluster. For example, *SAR0286* (*essC3* variant), *SAEMRSA15_02500* (*essC4* variant) and *SAPIG0304* (*essC2* variant) shared motifs associated with the CATH superfamily 1.20.5.170 [35]. This domain is characterised by single alpha-helices involved in coiled-coils or other helix-helix interfaces, is present in proteins from a wide range of organisms and is implicated in diverse functions. All six hypothetical proteins in the *essC2* variant shared a motif or some peptide sequence with a hypothetical protein in the *essC3* variant cluster.

### Module 3: a complex arrangement of predicted genes

Immediately downstream from Module 2 was a complex arrangement of CDSs that included a variable number of predicted transmembrane proteins, interspersed with a diverse arrangement of genes coding for hypothetical proteins of approximately 166 amino acids in length with a domain of unknown function named DUF600. These genes form Module 3, as illustrated in Fig. 1. The number of unique transmembrane proteins encoded in this region appeared to be small and was a combination of homologs of the following genes: *SAOUHSC_00270*; *SAOUHSC_00271*; *SAOUHSC_00272*; *SAOUHSC_00255* (which is sometimes found in this region downstream of *essC*, but in some isolates is located upstream of *esxA* where it is always found in association with *SAOUHSC_00254*); and a cluster of four genes which are always found together in the following combination: *SAEMRSA15_2510*, *SAEMRSA15_2520*, *SAEMRSA15_2530* and *SAEMRSA15_2540*.

The combination of these genes found in each genome varied between isolates. All strains had at least one homolog of these genes; no isolates possessed homologs of them all. Our analysis suggested that there may a relationship between the *essC* variant and the combination of genes in this region. For example, all *essC3* variants were associated with homologs of the genes *SAOUHSC_00272* and *SAOUHSC_00255*. Some, but not all, *essC3* variants were associated with the *SAEMRSA15_2510* to *SAEMRSA15_2540* cluster. None of the isolates with the *essC3* variant were associated with the *SAOUHSC_00271* gene. Different isolates within the same clonal complex appeared to possess the same combination of hypothetical transmembrane proteins encoded in Module 3. The exception was CC8, which had a wide variety of combinations. Some of the hypothetical transmembrane proteins were encoded in multiple copies in the same isolate. For example in the strain Newman, *NWMN_0230* encoded a protein with almost identical amino acid sequence to *NWMN_0237* (92 % amino acid identity; data not shown) [25].

The number of DUF600 genes present in representative of the different clonal complexes: the minimum number identified was two genes in CC22 isolates and the maximum number was 13 in CC8 isolates. The number of DUF600 genes also varied between isolates within the same clonal complex. In the *essC1* variant gene clusters, there was variation in the 3’ region of *essD*, the gene immediately upstream of the first DUF600 gene. Each *essD* variant was associated with a different downstream DUF600 protein variant, which broadly clustered into clonal complexes.

The relationship between this cluster of transmembrane and DUF600 proteins encoded at the *ess* locus is unclear. Several features support an association with the T7SS, including: i) these genes were always on the same coding strand, following immediately from Modules 1 and 2 described above; ii) there was little intergenic space between coding regions, suggesting that these genes may be under the same transcriptional control as other elements of the T7SS; iii) some of the genes encoded in this region shared sequence identity with genes that flanked known secretion component genes within the *ess* locus. For example, *SAOUHSC_00270* shared some sequence motifs with *SAOUHSC_00254* (21 % identity, 39 % similarity at the amino acid level) and *SAOUHSC_00271* (25 % identity, 52 % similarity at the amino acid level) with *SAOUHSC_00255*, genes that neighboured *SAOUHSC_00256* – the gene immediately upstream of *esxA. SAR0288*, part of the cluster of genes immediately downstream of *essC3* also shared 67 % amino acid sequence identity with *SAOUHSC_00254*.

### Module 4: two conserved hypothetical transmembrane proteins

Downstream of the highly variable Module 3 is a region that contains two genes (*SAOUHSC_00279* and *SAOUHSC_00280* represented as Module 4 in Fig. 1) that were conserved in all genomes investigated. Analysis of these two proteins revealed that they contained predicted N-terminal secretion signal sequences, and protein domains also found in proteins encoded elsewhere in the *ess* locus: *SAOUHSC_00279* contained a cystatin-like fold (DUF4467, Pfam accession PF14729) which was also present in *SAEMRSA_2520*, while *SAOUHSC_00280* harboured the DUF4064 domain (DUF4064, PF13273) which was also present in *SAEMRSA_2530*.

### Variable transcriptional landscape of the *ess* loci

**Fig. 3:**
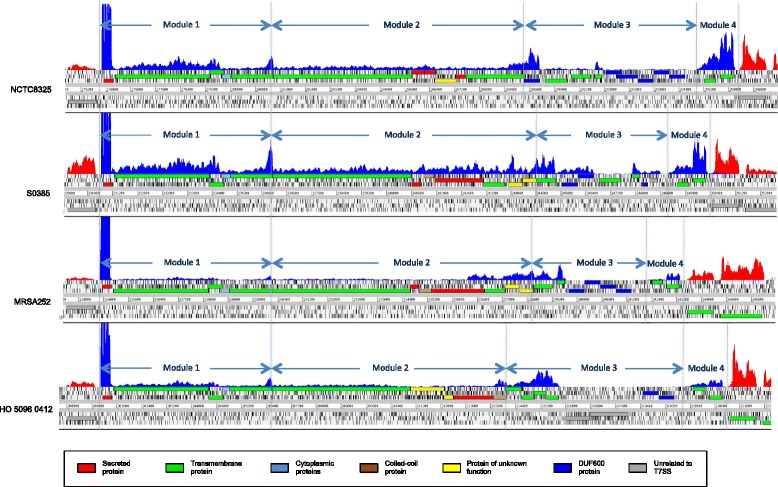
RNA-Seq profiles of the *ess* loci of four *S. aureus* reference isolates. Sequence reads from each of the four *S. aureus* reference isolates aligned to the respective *ess* loci viewed in Artemis. The mapped sequence read depth is displayed, with graphs representing the result of sequencing strand-specific cDNA mapping. The color of the graphed plots indicated the strand, with blue forward, and red reverse. CDSs from annotation are displayed in a six-frame translation, with the vertical black line indicating stop codons in frames. The coordinates in the figures indicate the position in the reference chromosome

RNA-Seq was used to examine the transcriptional profiles of the *ess* locus in the four *S. aureus* strains representative of the *essC* variants (NCTC 8325, S0835, MRSA252 and HO 5096 0412 as representatives of *essC1*, *essC2*, *essC3* and *essC4* variants, respectively) and, more specifically, to look for evidence of the expression of the hypothetical components of the *ess* loci identified in our analyses. Examination of the mapped strand-specific cDNA sequence data confirmed the gene prediction models of the *ess* regions, and revealed variation in the abundance of transcript across the *ess* region (Fig. 3). The highest level of transcript in the references strains was associated with the gene encoding the secreted effector protein EsxA of Module 1*,* a protein that has been found to play an important role in the pathogenesis of *S. aureus*, and also suggested as a potential target for vaccination [34]. This high level expression of *esxA* relative to other *ess* genes has been noted previously [14]. The rest of the genes in this module contained evidence of transcription, albeit at a lower level to that observed for *esxA*; in comparison to *esaA*, the next gene in the *ess* cluster, the transcript levels of *esxA* were between 60- and 70-fold greater in the four strains examined. The marked differences in transcript levels suggest separate promoters driving the expression of *esxA* and *esaA*. In their analysis of the RN6390 (a derivative of NCTC 8325) Kneuper et al. mapped the transcriptional start site upstream of *esxA*, and also identified a promoter in the *esxA*-*esaA* intergenic region [14]. RNA-Seq data supports this transcriptional organisation, with *esxA* and with the other components of the Module 1 (*esaA*, *essA*, *esaB*, *essB*) present on separate transcripts; *esxA* is present on a moncistronic transcript, and *esaA*, *essA*, *esaB*, *essB* on a polycistronic transcript that also contains components of Module 2, including *essC* and downstream CDSs.

**Fig. 4:**
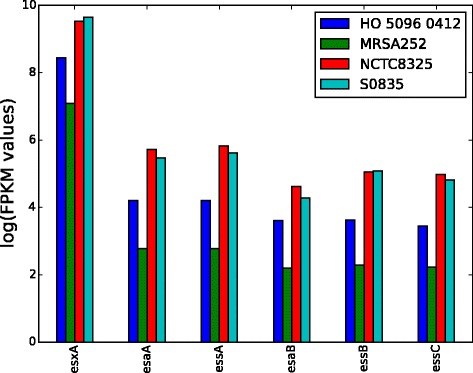
Relative expression of Module 1 components in *S. aureus* reference strains. RNA-Seq analysis of NCTC 8325, S0835, MRSA252 and HO 5096 0412, representatives of *essC1*, *essC2*, *essC3* and *essC4* variants respectively, was carried out to measure and compare the transcription of the Module 1 genes: *esxA*, *esaA*, *essA*, *esaB*, and *essB*. For each strain the mean fragments per kilobase of transcript per million mapped reads (FPKM) were calculated and normalized, using the expression value of the housekeeping gene *rpoD*. The normalized logFPKM values for each of the components of the Module 1 are plotted along with the values for *essC*, the first gene of Module 2 which is transcriptional linked to Modules 1 genes, *esaA*, *essA*, *esaB*, and *essB*

Comparison of the relative expression conserved components of the *ess* clusters revealed strain-specific differences in the levels of transcription (Fig. 4). The lowest expression was observed in MRSA252, followed by HO 5096 0412, with NCTC 8325 and then S0835 exhibiting the highest levels. For all the reference strains examined there was evidence of transcription of all of the Modules 2 and 4 genes (Additional file 1: Table S5). By contrast, not all of the genes found in the Module 3 regions exhibited evidence of transcription under the assay conditions used (Fig. 3), suggesting complex transcriptional controls in this region of the *ess* locus. Comparison of the gene organisation of the various Module 3 regions identified multiple intergenic regions; in contrast to the operonic organisation of Module 1 and 2 regions. Of the variable genes in this region of the *ess* loci those containing the DUF600 domain were the most variably expressed (Fig. 3; Additional file 1: Table S5). Notably each of the reference strains contained multiple copies of the DUF600 domain containing hypothetical proteins, raising the possibility that there may be functional redundancy associated with these proteins and, as a corollary of this, differential regulation.

### Evidence of recombination throughout the *ess* locus *in S. aureus*

**Fig. 5:**
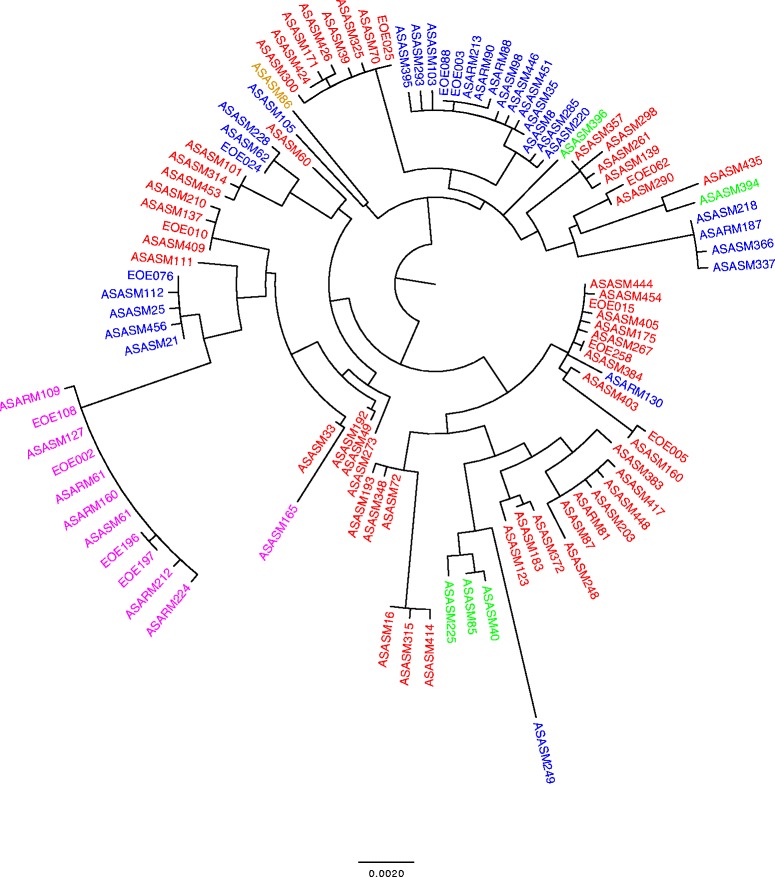
The distribution of *essC* variants across the *S. aureus* population. Maximum likelihood reconstruction of the phylogeny of 103 UKCRC *S. aureus* isolates based on MLST allele phylogeny, annotated by *essC* variant. Variant *essC1* is highlighted in red; *essC2* in green; *essC3* in blue and *essC4* in purple. The single variant where *essC* has been deleted is colored in ochre

Mapping the *essC* variants onto a *S. aureus* population framework generated from a phylogeny of MLST genes demonstrated that, although there is clustering of *essC* variants, there was also phylogenetic incongruence. This was suggestive of recombination at this locus across the species in a wide range of isolates (Fig. 5).

**Fig. 6:**
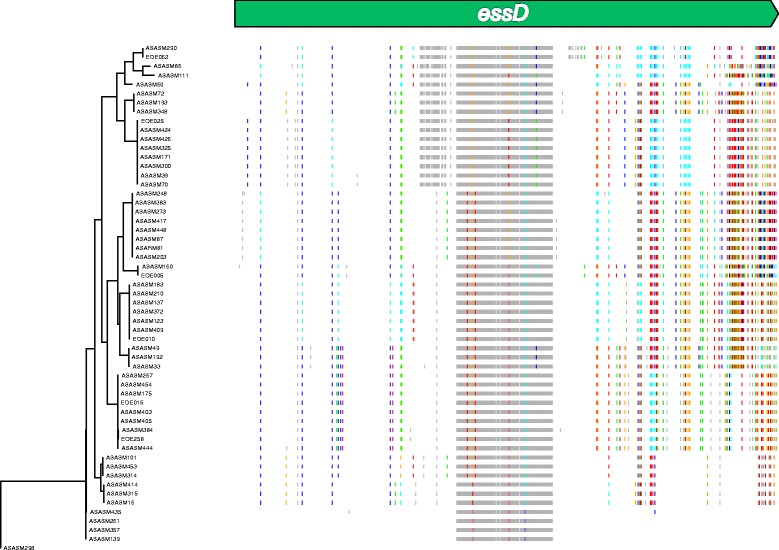
Evidence of recombination in the *essD* gene. On the left is a maximum likelihood phylogeny constructed with *essD* gene sequences from isolates containing the *essC1* subtype. To the right, each vertical colored line represents a SNP position in the *essD* sequence, top. Colored vertical lines along the tracks represent bases that differ from the ancestral sequence: grey = non-homoplastic change; colored lines represent homoplastic bases: red = A, blue = T, green = C, orange = G. The pattern of lines provide a barcode of *essD* similarity between strains and identifies regions containing homoplasies that are incongruent with the phylogeny and are associated with recombination

We applied further recombination detection methods using the Phi test within the PhiPack package [36] on individual genes within the *ess* locus. This method reported statistically significant *P* values for a number of genes in this locus, including those with a variable C-terminal region (*essC* (*P* = 0.0), *essD* (*P* = 0.0)). Although some conserved genes did not show evidence of recombination (e.g. *esxA*) other genes that are encoded in the conserved part of the ess locus did reach statistical significance using this method (*esaB* (*P* = 0.017), *essB* (*P* < 0.0001)). These indicate that the variation between the same gene in different isolates was more likely to have occurred by recombination than convergent selection or the random accumulation of homoplastic SNPs. To illustrate this, Fig. 6 shows the homoplastic SNPs in the *essD* genes of isolates with the *essC1* subtype, an apparent hotspot of recombination events within the *ess* operon.

### Components of the *ess* operon in other staphylococci

**Fig. 7:**
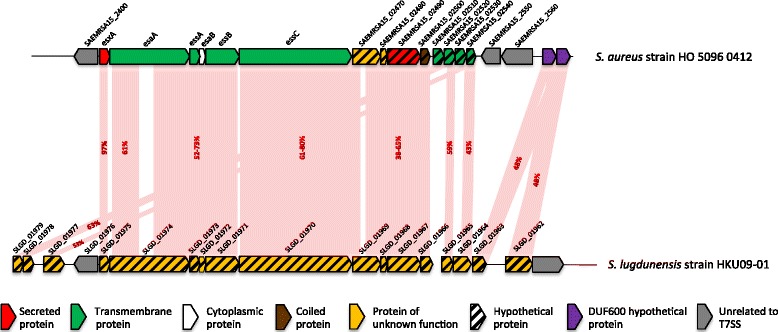
T7SS locus identified in *S. lugdunensis*. Comparison of the *ess* operon of *S. aureus* (strain HO 5096 0412) and *S. lugdunensis* (strain HKU09-01). Light pink shading demonstrates regions that are conserved between strains, with the degree of amino acid identity between strains marked in red

Comparative genomic analysis of *S. aureus* with publicly available reference genomes for other species in the *Staphylococcus* genus was conducted to investigate the wider distribution and genetic arrangement of this locus. As shown in Fig. 7, the *ess* locus was present in *Staphylococcus lugdunensis*, with homologs of the conserved portion of the locus from *esxA* to *essB* (*SLGD_01975* to *SLGD_01971*) and a gene similar to the *essC4* variant (*SLGD_01970*). There was also a cluster of genes similar to those downstream of *essC4*, including homologs of *SAEMRSA15_02470* to *SAEMRSA*_*02500*, *SAEMRSA_02520* and *SAEMRSA_02540* (*SLGD_01964* to *SLGD_01969*). There were two genes with homology to those coding for DUF600 proteins (*SLGD_01962* and *SLGD_01963*), but no obvious homologs of the two conserved genes found in Module 4 of the *ess* operon (*SAOUHSC_00279* and *SAOUHSC_00280*).

Of the other species examined within the *Staphylococcus* genus, none possessed the entire *ess* operon. However, *Staphylococcus pasteuri* contained one gene with sequence identity to the DUF600 gene (*STP1_1226*) and homologs of the cluster of four hypothetical transmembrane-protein coding genes, *SAEMRSA_02510* to *SAEMRSA_02540* (*STP1_1228* to *STP1_1231*). *Staphylococcus warneri* also possessed homologous genes to this cluster (*A284_00845* to *A284_00860*). None of the genes identify in these staphylococci exhibited high DNA identity to *S. aureus* homologs suggestive of interspecies recombination. A complete list of the species and isolates examined can be found in Additional file 1: Table S3.

## Discussion

In this study we have described, for the first time, the organisational variation in the *ess* locus using *S. aureus* isolates from a broad range of clonal complexes from two distinct genome collections. We identified genes conserved across all isolates and characterised variants in the *ess* operon, including a number of further candidate substrate proteins that may be secreted by the *S. aureus* T7SS.

Despite the variation elsewhere in the *ess* locus, it was striking that the cluster of genes in Module 1, from *esxA* to *essB*, was conserved among almost all isolates, suggesting a strong survival benefit or functional constraint on this cluster. In-frame deletions of each of these genes have been shown to abolish secretion of T7SS substrate proteins, supporting the assertion that these genes encode core components of the T7SS [13, 14, 19].

A key finding in this study was the identification of four variants of the *essC* gene. This membrane-bound protein is essential for secretion of all effector proteins in the T7SS [13, 20]. Variation within this gene occurred in the region encoding the C-terminus of EssC, covering the final ATPase domain. We have shown that each *essC* variant was associated with a unique cluster of downstream genes, suggesting that the EssC C-terminus may play a role in interacting with the proteins encoded immediately downstream from *essC*. One of the proteins encoded downstream of each EssC variant is from the WXG superfamily, and in the commonly studied *S. aureus* Newman, USA300 and RN6390 strains it is EsxB. Studies have shown that *esxB* is expressed at a very similar level to *essC* [14], and structural analysis with the related EccC protein of Actinobacteria has shown that EsxB binds to the C-terminal ATPase domain of the EssC homolog and promotes its multimerisation [8]. We postulate that the WXG protein encoded downstream of each *essC* variant interacts with the C-terminal domain of its cognate EssC protein to activate secretion. It is also possible that this sequence-variable EssC domain may recognise variant-specific substrate proteins or accessory factors. While the genetic basis of this variation and its functional consequence remains unclear, the arrangement of genes and their incongruence with MLST suggest that recombination has played a role in the evolution of this locus. Comparative genomic analysis of *S. aureus* has revealed that recombination in the core genome is relatively rare [37]. It is therefore notable that the T7SS undergoes such a degree of recombination and suggests that the recombination-driven generation of variation may be an important system for the biology of the organism.

The function of many of the proteins in Module 2, downstream from *essC*, remains unclear. The best characterised cluster was downstream from *essC1*, which has been studied in a small number of CC8 isolates and includes genes encoding the secreted proteins EsxB, EsxC and EsxD and the transmembrane protein EssD. These proteins appear to have a functional relationship, with reports that strains lacking each of these proteins affect the stability or expression of the others, while EsxB and EsxD have been shown to form heterodimers [20]. Further work is required to determine whether a similar relationship is present between proteins encoded in the other *ess* variants.

Although there is a limit to information that can be derived from the bioinformatics analysis of four variants of Module 2 identified in this study, the observation that different variants encoded proteins that shared sequence motifs, particularly the modules associated with *essC3* and *essC2*, implied some redundancy in their function. However, there were also marked differences between variants. For example, the *essC2*, *essC3* and *essC4* variants all contained a hypothetical protein with the WXG motif (SAPIG0305, SAR0287, SAEMRSA15_02490 respectively) that was much larger (440–556 aa) than the previously identified T7SS substrates associated with *essC1* (for example EsxB and EsxD are 104 and 105 amino acids in length respectively). There was also no transmembrane protein of a comparable size to EssD encoded in the other variants.

The association between EssD and the DUF600 hypothetical proteins has been observed previously, with the C-terminus of some EssD sequences in *S. aureus* (as well as the EssD-like protein in *B. subtilis*, YeeE) containing the DUF600 motif [21]. We observed that there was a relationship between the C-terminal sequence of EssD and the immediately downstream DUF600 protein. However, the molecular relationship between these two proteins is unclear. We have also identified a range of isolates that did not possess the *essD* gene, which raised the question of whether other genes in these variants serve an equivalent function.

Previous evidence has suggested that there is very little variation within coding regions between isolates belonging to the same lineage [38]. However, we have shown that the copy number of DUF600 proteins varied between isolates of the same clonal complex, and also there is differential expression of these proteins within *ess* clusters. A recent study by Baek and colleagues demonstrated that the greatest sequence variation between strains of the same *S. aureus* lineage (NCTC 8325) occurred between homologs of the DUF600 proteins [39]. This included variation in the copy number of DUF600 proteins encoded in each genome as well as the sequence of each copy. The reason for this variation is unknown, but the fact that it occurs between such closely related lineages suggests considerable selection pressure on these genes.

There are many hypothetical transmembrane proteins encoded within Module 3 in the *ess* operon, associated with the DUF600 proteins. Their function and relationship to the T7SS will require further investigation. The presence of these hypothetical transmembrane proteins without the remainder of the T7SS apparatus in the genomes of *S. warneri* and *S. pasteuri* may suggest they are not key components of this system. However it is unclear why these genes are located in the same region of the *S. aureus* genome, often flanked by DUF600 proteins.

The T7SS has been described in other Gram positive bacteria [2, 3, 6, 7]. We describe here homologs of a large number of genes encoding this system in *S. lugdunensis*, a medium-pathogenic coagulase-negative staphylococcus which shares a number of clinical features with *S. aureus*, including the ability to form abscesses [40]. The mechanisms of virulence of this organism are poorly understood, but it is possible that, as with *S. aureus*, the T7SS could have a role in abscess development and therefore in pathogenicity. Investigation into a broader range of staphylococcal isolates may provide clues as to the evolution of the *ess* locus.

## Conclusions

Having described a range of hypothetical proteins that will require further molecular research to determine their role and function, our study provides further avenues for investigation. Recent studies have suggested that the expression of key genes of the T7SS, including *esxA*, are under complex regulatory control and vary between closely related isolates in the same clonal complex [14]. In this study we have demonstrated a far greater diversity of genetic organisation of the *ess* operon, which is likely to have implications for gene regulation in the broader range of clonal complexes. Mutants in the T7SS have impaired ability to persist in the complex interplay between host and pathogen during colonisation and abscess formation [14, 41]. However, published research has focused on a narrow range of *S. aureus* isolates, all of which possess the *essC1* operon arrangement. It is unclear what effect other arrangements will have on the pathogenicity of *S. aureus*, or its abscess-forming phenotype.

## Methods

### Bacterial isolates and whole genome sequencing

*S. aureus* whole genome sequence data utilized in this study was generated by the Wellcome Trust Sanger Institute (WTSI). The *S. aureus* isolates were obtained from human bacteremia cases at Addenbrooke’s Hospital, Cambridge, UK between 2006 and 2012. Further stored isolates from neighbouring hospitals in the East of England, collected between 1998 and 2011, were also included. From this broad collection, 103 isolates were chosen to represent as many different clonal complex (CC) and MLST types (ST) as possible, to provide a snapshot of the genetic diversity of disease causing isolates from this large scale UK collection. To augment the collection, a further 50 published and publicly available *S. aureus* sequences from the European Bioinformatics Institute were included in the analysis. A full list of isolates used in this study, along with accession numbers, is presented in Additional file 1: Tables S1, S2 and S3. All samples were sequenced as multiplexed libraries using the Illumina HiSeq 2000 analyzers as previously described [23].

### Ethics

Written informed consent from patients was not required as all bacterial isolates were collected, processed and stored as part of routine clinical care. The study protocol was approved by the National Research Ethics Service (reference 11/EE/0499), and by the Cambridge University Hospitals NHS Foundation Trust Research and Development Department (reference A092428).

### Transcriptional analyses

Strains of *S. aureus* (Newman, S0385, MRSA252 and HO 5096 0412 reference genomes strains) were incubated shaking at 200 rpm for 16 h at 37 °C. The overnight culture was used to inoculate 25 ml TSB media to an OD600 of 0.03. 2 ml of OD600 1.0 cells were harvested by centrifugation at 13000 g for 2 min and processed for RNA extraction using the Ambion Ribo-PureTM Bacteria kit as per the manufacturers’ guidance. Three biological and three technical replicates were performed for each strain. The RNA quality and concentration was assessed by Agilent BionalyserTM, NanodropTM and visually on a 1 % agarose gel containing 1 % gel red (Biotium) and visualised by gel doc imaging system (Bio-Rad).

For RNA-Seq, total RNA was reverse transcribed using SuperScript III reverse transcriptase (Invitrogen). Actinomycin D (6 μg/ml, Sigma) was added to the reaction to avoid spurious second-strand cDNA synthesis [35]. cDNA was purified using QIAquick PCR purification kit (Qiagen) and used for single stranded cDNA library construction as previously [25, 36]. FRT-seq Illumina libraries were constructed as previously described [37]. RNA sequencing was performed using an Illumina HiSeq 2000 sequencer, and the reads processed as previously described [38].

### Bioinfomatic analyses

Assemblies were created with Velvet v1.2.09 [24] using the VelvetOptimiser.pl v2.2.5 (http://www.vicbioinformatics.com/software.velvetoptimiser.shtml) script to optimize the kmer length. Automatic annotation of the Accessory genome contigs was carried out using PROKKA [25]. Detailed comparisons of individual sequences were conducted on the *de novo* assemblies using BlastN [26], and was facilitated by using the Artemis Comparison Tool (ACT) [27].

Individual T7SS genes were identified from the publicly available Newman strain reference genome (the most commonly studied *S. aureus* strain in the T7SS literature) [28]. Primers were designed for the terminal 30 nucleotides of each of these genes. *In silico* PCR using these primers was performed on the *de novo* assemblies for all isolates using a Python script, to determine the presence or absence of each gene in each isolate. In brief, the script used approximate regular expressions to identify all best matches to each primer sequence in each genome, allowing up to 3 mismatches per match. Where forward and reverse primers were in the correct orientation relative to each other and produced a product of approximately the correct length (defined as <10 kb), products were extracted as fasta files. If a gene could not be identified in an isolate using this method, tblastx comparisons [26] were generated comparing the novel isolate with the reference. This comparison was then visualised graphically using the Artemis Comparison Tool [27] to confirm the absence of the gene or identify major variants in the *ess* locus. *In silico* primers were developed for all novel genes identified and the *in silico* PCR process repeated until all genes in the *ess* locus had been identified. All primers used in this analysis are presented in Additional file 1: Table S4.

Bioinformatic searches on all genes and putative gene products were conducted using the web-resources NCBI BLASTn [29]) (available at www.ncbi.nlm.nih.gov/blast/), and Pfam [30] (available at http://pfam.xfam.org/), InterPro [31] (available at http://www.ebi.ac.uk/interpro/). Maximum likelihood phylogenetic trees were generated for whole genome sequences of the 103 isolates sequenced using RAxML [33]. Further maximum-likelihood phylogenetic trees based on single genes or clusters of genes in the *ess* locus were generated using PhyML 3.1 [34].

For the RNA-Seq data samples were mapped to the appropriate reference genomes, and transcripts were assembled, and expression values for the assembled genes and transcripts were computed. Reads were aligned using the Tophat aligner [40]. One of the sequence samples in the MRSA252 strain RNA-Seq experiments produced a very poor yield, therefore this was excluded from the rest of the analysis. After merging the biological with the corresponding technical aligned samples, transcriptome assembly for each sample in the strains was carried out using cufflinks [41]. To acquire a single trasncriptome for each strain, we merged the three assemblies produced by cufflinks and we quantified the abundances of each sample using cuffquant. Differential expression (cuffdiff) as well as normalization (cuffnorm) within the samples of each strain was carried out to acquire the expression values of assembled genes and transcripts.

To compare the expression levels of the genes of interest (*esxA*, *esaA*, *essA*, *esaB*, *essB* and *essC*) in the different strains and remove any extra source of bias in the data, we performed an extra normalization, using the expression value of a housekeeping gene, which is *rpoD* in our case. More specifically, we scaled the fragments per kilobase of transcript per million (FPKM) mapped reads values of the housekeeping gene across the four strains (**h**ousekeeping for the specific **s**train, *h*_*s*_) and we resized the FPKM values of all the genes based on the *h*_*s*_.

### Availability of supporting data

All sequences from this study have been submitted to the European Nucleotide Archive (ENA; http://www.ebi.ac.uk/ena) under the study numbers ERP000871, ERP001009, and ERP001320; individual accession numbers are given in Additional file 1: Table S1. RNA-Seq data has been submitted to the European Nucleotide Archive with accession number ERP009279 and in Array express under accession number E-ERAD-362.

## Additional file

Additional file 1:
**Table S1.** List of all whole genome sequenced *S. aureus* isolates used in this study. **Table S2.** List of publicly available reference strains of *S. aureus* used in this study. **Table S3.** List of publicly available genomes of reference strains of non-*S. aureus* staphylococci analysed in this study*.* **Table S4.** List of primer sequences used for *in silico* PCR analysis. **Table S5.** RNA-Seq transcriptional analysis of the *ess* clusters of four reference genomes strains of *S. aureus*. (DOC 405 kb)

## Abbreviations

CC: clonal complex
CDS: protein coding sequence
DUF: domain of unknown function
MLST: multi-locus sequence type
RNA-Seq: RNA sequencing
T7SS: Type VII protein secretion

## References

[1] Costa TRD, Felisberto-Rodrigues C, Meir A, Prevost MS, Redzej A, Trokter M (2015). Secretion systems in Gram-negative bacteria: structural and mechanistic insights. Nat Rev Micro.

[2] Abdallah AM, Gey van Pittius NC, Champion PAD, Cox J, Luirink J, Vandenbroucke-Grauls CMJE (2007). Type VII secretion--mycobacteria show the way. Nat Rev Micro.

[3] Pallen MJ (2002). The ESAT-6/WXG100 superfamily -- and a new Gram-positive secretion system?. Trends Microbiol.

[4] Schneewind O, Missiakas DM (2012). Protein secretion and surface display in Gram-positive bacteria. Philos Trans R Soc Lond B Biol Sci.

[5] Majlessi L, Prados-Rosales R, Casadevall A, Brosch R (2015). Release of mycobacterial antigens. Immunol Rev.

[6] Hsu T, Hingley-Wilson SM, Chen B, Chen M, Dai AZ, Morin PM (2003). The primary mechanism of attenuation of bacillus Calmette-Guerin is a loss of secreted lytic function required for invasion of lung interstitial tissue. Proc Natl Acad Sci U S A.

[7] Houben D, Demangel C, van Ingen J, Perez J, Baldeon L, Abdallah AM (2012). ESX-1-mediated translocation to the cytosol controls virulence of mycobacteria. Cell Microbiol.

[8] Rosenberg OS, Dovala D, Li X, Connolly L, Bendebury A, Finer-Moore J (2015). Substrates control multimerization and activation of the multi-domain ATPase motor of type VII secretion. Cell.

[9] Poulsen C, Panjikar S, Holton SJ, Wilmanns M, Song Y-H (2014). WXG100 protein superfamily consists of three subfamilies and exhibits an α-helical C-terminal conserved residue pattern. PLoS One.

[10] Sysoeva TA, Zepeda-Rivera MA, Huppert LA, Burton BM (2014). Dimer recognition and secretion by the ESX secretion system in *Bacillus subtilis*. Proc Natl Acad Sci U S A.

[11] Baptista C, Barreto HC, São-José C (2013). High levels of DegU-P activate an Esat-6-like secretion system in *Bacillus subtilis*. PLoS One.

[12] Garufi G, Butler E, Missiakas D (2008). ESAT-6-like protein secretion in *Bacillus anthracis*. J Bacteriol.

[13] Burts ML, Williams WA, DeBord K, Missiakas DM (2005). EsxA and EsxB are secreted by an ESAT-6-like system that is required for the pathogenesis of *Staphylococcus aureus* infections. Proc Natl Acad Sci U S A.

[14] Kneuper H, Cao ZP, Twomey KB, Zoltner M, Jäger F, Cargill JS (2014). Heterogeneity in *ess* transcriptional organization and variable contribution of the Ess/Type VII protein secretion system to virulence across closely related *Staphylocccus aureus* strains. Mol Microbiol.

[15] Lowy FD (1998). *Staphylococcus aureus* Infections. N Engl J Med.

[16] Klevens RM, Morrison MA, Nadle J, Petit S, Gershman K, Ray S (2007). Invasive methicillin-resistant *Staphylococcus aureus* infections in the United States. Jama.

[17] Moran GJ, Krishnadasan A, Gorwitz RJ, Fosheim GE, McDougal LK, Carey RB (2006). Methicillin-resistant *S. aureus* infections among patients in the emergency department. N Engl J Med.

[18] Windmuller N, Witten A, Block D, Bunk B, Sproer C, Kahl BC (2015). Transcriptional adaptations during long-term persistence of *Staphylococcus aureus* in the airways of a cystic fibrosis patient. Int J Med Microbiol.

[19] Burts ML, DeDent AC, Missiakas DM (2008). EsaC substrate for the ESAT-6 secretion pathway and its role in persistent infections of *Staphylococcus aureus*. Mol Microbiol.

[20] Anderson M, Aly KA, Chen Y-H, Missiakas D (2013). Secretion of atypical protein substrates by the ESAT-6 secretion system of *Staphylococcus aureus*. Mol Microbiol.

[21] Anderson M, Chen Y-H, Butler EK, Missiakas DM (2011). EsaD, a secretion factor for the Ess pathway in *Staphylococcus aureus*. J Bacteriol.

[22] Enright MC, Robinson DA, Randle G, Feil EJ, Grundmann H, Spratt BG (2002). The evolutionary history of methicillin-resistant *Staphylococcus aureus* (MRSA). Proc Natl Acad Sci.

[23] Lindsay JA (2014). *Staphylococcus aureus* genomics and the impact of horizontal gene transfer. Int J Med Microbiol.

[24] Tanaka Y, Kuroda M, Yasutake Y, Yao M, Tsumoto K, Watanabe N (2007). Crystal structure analysis reveals a novel forkhead-associated domain of ESAT-6 secretion system C protein in *Staphylococcus aureus*. Proteins.

[25] Baba T, Bae T, Schneewind O, Takeuchi F, Hiramatsu K (2008). Genome sequence of S*taphylococcus aureus* strain Newman and comparative analysis of staphylococcal genomes: polymorphism and evolution of two major pathogenicity islands. J Bacteriol.

[26] Diep BA, Gill SR, Chang RF, Phan TH, Chen JH, Davidson MG (2006). Complete genome sequence of USA300, an epidemic clone of community-acquired meticillin-resistant *Staphylococcus aureus*. Lancet.

[27] Gill SR, Fouts DE, Archer GL, Mongodin EF, Deboy RT, Ravel J (2005). Insights on evolution of virulence and resistance from the complete genome analysis of an early methicillin-resistant *Staphylococcus aureus* strain and a biofilm-producing methicillin-resistant *Staphylococcus epidermidis* strain. J Bacteriol.

[28] Kuroda M, Ohta T, Uchiyama I, Baba T, Yuzawa H, Kobayashi I (2001). Whole genome sequencing of meticillin-resistant *Staphylococcus aureus*. Lancet.

[29] Baba T, Takeuchi F, Kuroda M, Yuzawa H, Aoki K-I, Oguchi A (2002). Genome and virulence determinants of high virulence community-acquired MRSA. Lancet.

[30] Holden MTG, Feil EJ, Lindsay JA, Peacock SJ, Day NPJ, Enright MC (2004). Complete genomes of two clinical *Staphylococcus aureus* strains: evidence for the rapid evolution of virulence and drug resistance. Proc Natl Acad Sci U S A.

[31] Holden MTG, Lindsay JA, Corton C, Quail MA, Cockfield JD, Pathak S (2010). Genome sequence of a recently emerged, highly transmissible, multi-antibiotic- and antiseptic-resistant variant of methicillin-resistant *Staphylococcus aureus*, sequence type 239 (TW). J Bacteriol.

[32] Holden MTG, Hsu L-Y, Kurt K, Weinert LA, Mather AE, Harris SR (2013). A genomic portrait of the emergence, evolution, and global spread of a methicillin-resistant *Staphylococcus aureus* pandemic. Genome Res.

[33] Schijffelen MJ, Boel CH, van Strijp JA, Fluit AC (2010). Whole genome analysis of a livestock-associated methicillin-resistant *Staphylococcus aureus* ST398 isolate from a case of human endocarditis. BMC Genomics.

[34] Champion PAD, Champion MM, Manzanillo P, Cox JS (2009). ESX-1 secreted virulence factors are recognized by multiple cytosolic AAA ATPases in pathogenic mycobacteria. Mol Microbiol.

[35] Sillitoe I, Cuff AL, Dessailly BH, Dawson NL, Furnham N, Lee D (2013). New functional families (FunFams) in CATH to improve the mapping of conserved functional sites to 3D structures. Nucleic Acids Res.

[36] Bruen TC, Philippe H, Bryant D (2006). A simple and robust statistical test for detecting the presence of recombination. Genetics.

[37] Castillo-Ramírez S, Corander J, Marttinen P, Aldeljawi M, Hanage WP, Westh H (2012). Phylogeographic variation in recombination rates within a global clone of methicillin-resistant *Staphylococcus aureus*. Genome Biol.

[38] Lindsay JA (2010). Genomic variation and evolution of *Staphylococcus aureus*. Int J Med Microbiol.

[39] Bæk KT, Frees D, Renzoni A, Barras C, Rodriguez N, Manzano C (2013). Genetic variation in the *Staphylococcus aureus* 8325 strain lineage revealed by whole-genome sequencing. PLoS One.

[40] Frank KL, Del Pozo JL, Patel R (2008). From clinical microbiology to infection pathogenesis: how daring to be different works for *Staphylococcus lugdunensis*. Clin Microbiol Rev.

[41] Cheng AG, DeDent AC, Schneewind O, Missiakas D (2011). A play in four acts: *Staphylococcus aureus* abscess formation. Trends Microbiol.

