# Supplementary material for: The Ess/Type VII secretion system of Staphylococcus aureus shows unexpected genetic diversity
Source: BMC Genomics. 2016 Mar 11;17:222. doi: 10.1186/s12864-016-2426-7 (PMC4788903; doi:10.1186/s12864-016-2426-7)
Supplement: Additional file 1: — Table S1. List of all whole genome sequenced S. aureus isolates used in this study. Table S2. List of publicly available reference strains of S. aureus used in this study. Table S3. List of publicly available genomes of reference strains of non-S. aureus staphylococci analysed in this study. Table S4. List of primer sequences used for in silico PCR analysis. Table S5. RNA-Seq transcriptional analysis of the ess clusters of four reference genomes strains of S. aureus. (DOC 405 kb) [file 12864_2016_2426_MOESM1_ESM.doc]

**Additional file 1**

**Table S1** – List of whole genome sequenced *S. aureus* isolates used in this study, with the sequence accession numbers, associated strain type (ST), clonal complex (CC) and *essC* variant identified during this study*.* Where an isolate was predicted to belong to a novel ST, the nearest ST is stated along with the unknown allele where it is a single locus variant (SLV).

| **Accession Number** | **Isolate Name** | **ST** | **CC** | ***essC* variant** |
| --- | --- | --- | --- | --- |
| ERS050056 | ASARM81 | 1 | CC1 | 1 |
| ERS072042 | ASASM87 | 1 | CC1 | 1 |
| ERS133854 | ASASM448 | 81 | CC1 | 1 |
| ERS072189 | ASASM248 | 1207 | CC1 | 1 |
| ERS133870 | ASASM383 | 1278 | CC1 | 1 |
| ERS072426 | ASASM203 | pta_SLV_ST1 | CC1 | 1 |
| ERS133823 | ASASM417 | tpi_SLV_ST1 | CC1 | 1 |
| ERS071988 | ASASM21 | 12 | CC12 | 3 |
| ERS108245 | EOE076 | 12 | CC12 | 3 |
| ERS072304 | ASASM25 | 1460 | CC12 | 3 |
| ERS133863 | ASASM456 | gmk_SLV_ST12 | CC12 | 3 |
| ERS072328 | ASASM112 | tpi_SLV_ST12 | CC12 | 3 |
| ERS072414 | ASASM85 | 15 | CC15 | 2 |
| ERS072167 | ASASM225 | 582 | CC15 | 2 |
| ERS072005 | ASASM40 | arcC_SLV_ST15 | CC15 | 2 |
| ERS072026 | ASASM62 | 188 | CC188 | 3 |
| ERS108458 | EOE024 | 188 | CC188 | 3 |
| ERS072170 | ASASM228 | pta_SLV_ST188 | CC188 | 3 |
| ERS050037 | ASARM61 | 22 | CC22 | 4 |
| ERS050124 | ASARM160 | 22 | CC22 | 4 |
| ERS072315 | ASASM61 | 22 | CC22 | 4 |
| ERS072333 | ASASM127 | 22 | CC22 | 4 |
| ERS108173 | EOE002 | 22 | CC22 | 4 |
| ERS108485 | EOE108 | 22 | CC22 | 4 |
| ERS050171 | ASARM212 | 1784 | CC22 | 4 |
| ERS134155 | ASARM224 | 2046 | CC22 | 4 |
| ERS108509 | EOE196 | gmk_SLV_ST22 | CC22 | 4 |
| ERS108350 | EOE197 | gmk_SLV_ST22 | CC22 | 4 |
| ERS050080 | ASARM109 | tpi_SLV_ST22 | CC22 | 4 |
| ERS071984 | ASASM16 | 25 | CC25 | 1 |
| ERS072245 | ASASM315 | 26 | CC25 | 1 |
| ERS133820 | ASASM414 | aroE_SLV_ST25 | CC25 | 1 |
| ERS072000 | ASASM35 | 30 | CC30 | 3 |
| ERS072300 | ASASM8 | 34 | CC30 | 3 |
| ERS050065 | ASARM90 | 36 | CC30 | 3 |
| ERS050172 | ASARM213 | 36 | CC30 | 3 |
| ERS108174 | EOE003 | 36 | CC30 | 3 |
| ERS108257 | EOE088 | 36 | CC30 | 3 |
| ERS072416 | ASASM103 | 39 | CC30 | 3 |
| ERS072434 | ASASM285 | arcC_SLV_ST34 | CC30 | 3 |
| ERS072228 | ASASM293 | arcC_yqil_DLV_ST30 | CC30 | 3 |
| ERS072053 | ASASM98 | aroE_SLV_ST30 | CC30 | 3 |
| ERS133891 | ASASM446 | glpF_SLV_ST30 | CC30 | 3 |
| ERS133874 | ASASM395 | pta_SLV_ST30 | CC30 | 3 |
| ERS072361 | ASASM220 | pta_SLV_ST34 | CC30 | 3 |
| ERS133857 | ASASM451 | yqil_SLV_ST30 | CC30 | 3 |
| ERS050063 | ASARM88 | yqil_SLV_ST36 | CC30 | 3 |
| ERS072032 | ASASM70 | 45 | CC45 | 1 |
| ERS108197 | EOE025 | 45 | CC45 | 1 |
| ERS133830 | ASASM424 | 47 | CC45 | 1 |
| ERS072234 | ASASM300 | 54 | CC45 | 1 |
| ERS072391 | ASASM325 | 508 | CC45 | 1 |
| ERS072410 | ASASM39 | glpF_SLV_ST45 | CC45 | 1 |
| ERS072423 | ASASM171 | tpi_SLV_ST47 | CC45 | 1 |
| ERS133832 | ASASM426 | yqil_SLV_ST47 | CC45 | 1 |
| ERS108543 | EOE010 | 5 | CC5 | 1 |
| ERS133815 | ASASM409 | 5 | CC5 | 1 |
| ERS072358 | ASASM210 | tpi_SLV_ST5 | CC5 | 1 |
| ERS072336 | ASASM137 | yqil_SLV_ST5 | CC5 | 1 |
| ERS072201 | ASASM261 | 51 | CC51 | 1 |
| ERS072090 | ASASM139 | 95 | CC51 | 1 |
| ERS072285 | ASASM357 | 121 | CC51 | 1 |
| ERS072384 | ASASM298 | 123 | CC51 | 1 |
| ERS050151 | ASARM187 | 59 | CC59 | 3 |
| ERS072161 | ASASM218 | 59 | CC59 | 3 |
| ERS072458 | ASASM337 | 1224 | CC59 | 3 |
| ERS072294 | ASASM366 | glpF_SLV_ST59 | CC59 | 3 |
| ERS072014 | ASASM49 | 7 | CC7 | 1 |
| ERS072137 | ASASM192 | 789 | CC7 | 1 |
| ERS108186 | EOE015 | 8 | CC8 | 1 |
| ERS133850 | ASASM444 | 8 | CC8 | 1 |
| ERS050100 | ASARM130 | 241 | CC8 | 3 |
| ERS133859 | ASASM454 | 609 | CC8 | 1 |
| ERS133809 | ASASM403 | 630 | CC8 | 1 |
| ERS133811 | ASASM405 | 2021 | CC8 | 1 |
| ERS133790 | ASASM384 | 2416 | CC8 | 1 |
| ERS108407 | EOE258 | 2416 | CC8 | 1 |
| ERS072204 | ASASM267 | pta_SLV_ST8 | CC8 | 1 |
| ERS072125 | ASASM175 | tpi_SLV_ST8 | CC8 | 1 |
| ERS072388 | ASASM314 | 78 | CC88 | 1 |
| ERS072056 | ASASM101 | 88 | CC88 | 1 |
| ERS134092 | ASASM453 | pta_SLV_ST88 | CC88 | 1 |
| ERS072448 | ASASM183 | 109 | CC9 | 1 |
| ERS072075 | ASASM123 | tpi_SLV_ST9 | CC9 | 1 |
| ERS134083 | ASASM372 | yqil_SLV_ST109 | CC9 | 1 |
| ERS072318 | ASASM72 | 97 | CC97 | 1 |
| ERS072276 | ASASM348 | 464 | CC97 | 1 |
| ERS072138 | ASASM193 | tpi_SLV_ST97 | CC97 | 1 |
| ERS072210 | ASASM273 | 6 | unknown_CC | 1 |
| ERS072060 | ASASM105 | 10 | unknown_CC | 3 |
| ERS072345 | ASASM165 | 20 | unknown_CC | 4 |
| ERS072306 | ASASM33 | 72 | unknown_CC | 1 |
| ERS072024 | ASASM60 | 101 | unknown_CC | 1 |
| ERS072225 | ASASM290 | 130 | unknown_CC | 1 |
| ERS072041 | ASASM86 | 182 | unknown_CC | Absent |
| ERS133841 | ASASM435 | 291 | unknown_CC | 1 |
| ERS108176 | EOE005 | 361 | unknown_CC | 1 |
| ERS133800 | ASASM394 | 398 | unknown_CC | 2 |
| ERS072110 | ASASM160 | 672 | unknown_CC | 1 |
| ERS133802 | ASASM396 | 718 | unknown_CC | 2 |
| ERS072063 | ASASM111 | 1035 | unknown_CC | 1 |
| ERS108230 | EOE062 | DLV_ST130 | unknown_CC | 1 |
| ERS072370 | ASASM249 | unknown_ST | unknown_CC | 3 |

**Table S2** *–* List of publicly available reference strains of *S. aureus* used in this study, with sequence accession number, associated strain type (ST), clonal complex (CC) and *essC* variant identified during this study*.*

| **Strain** | **Accession Number** | **ST** | **CC** | ***essC* variant** |
| --- | --- | --- | --- | --- |
| MW2 | BA000033 | 1 | 1 | 1 |
| MSSA476 | BX571857 | 1 | 1 | 1 |
| Mu3 | AP009324 | 5 | 5 | 1 |
| Mu50 | BA000017 | 5 | 5 | 1 |
| N315 | BA000018 | 5 | 5 | 1 |
| ED98 | CP001781 | 5 | 5 | 1 |
| JH9 | CP000703 | 105 | 5 | 1 |
| JH1 | CP000736 | 105 | 5 | 1 |
| Newman | AP009351 | 8 | 8 | 1 |
| NCTC 8325 | CP000253 | 8 | 8 | 1 |
| USA300 FRP3757 | CP000255 | 8 | 8 | 1 |
| USA300 TCH1516 | CP000730 | 8 | 8 | 1 |
| COL | CP000046 | 250 | 8 | 1 |
| HO 5096 0412 | HE681097 | 22 | 22 | 4 |
| TCH60 | CP002110 | 30 | 30 | 3 |
| 55/2053 | CP002388 | 30 | 30 | 3 |
| MRSA252 | BX571856 | 36 | 30 | 3 |
| CA-347 | CP006044 |  | 45 | 1 |
| MSHR1132 | FR821777 |  | 75 | 1 |
| ED133 | CP001996 |  | 133 | 1 |
| RF122 | AJ938182 | 151 | 151 | 1 |
| 04-02981 | CP001844 |  | 225 | 1 |
| JKD6008 | CP002120 | 239 | 239 | 3 |
| TW20 | FN433596 | 239 | 239 | 3 |
| ST398 | AM990992 | 398 | 398 | 2 |
| LGA 251 | FR821779 | 425 | 425 | 3 |
| M1 | HF937103 | 8 |  | 1 |
| 6850 | CP006706 | 50 |  | 1 |
| M013 | CP003166 | 59 |  | 3 |
| SA957 | CP003603 | 59 |  | 3 |
| SA40 | CP003604 | 59 |  | 3 |
| CN1 | CP003979 | 72 |  | 1 |
| 11819-97 | CP003194 | 80 |  | 1 |
| JKD6159 | CP002114 | 93 |  | 1 |
| ST228 isolate 10388 | HE579059 | 228 |  | 1 |
| ST228 isolate 10497 | HE579061 | 228 |  | 1 |
| ST228 isolate 15532 | HE579063 | 228 |  | 1 |
| ST228 isolate 16035 | HE579065 | 228 |  | 1 |
| ST228 isolate 16125 | HE579067 | 228 |  | 1 |
| ST228 isolate 18341 | HE579069 | 228 |  | 1 |
| ST228 isolate 18412 | HE579071 | 228 |  | 1 |
| ST228 isolate 18583 | HE579073 | 228 |  | 1 |
| T0131 | CP002643 | 239 |  | 3 |
| Bmb9393 | CP005288 | 239 |  | 3 |
| Z172 | CP006838 | 239 |  | 3 |
| 71193 | CP003045 | 398 |  | 2 |
| 08BA02176 | CP003808 | 398 |  | 2 |
| VC40 | CP003033 |  |  | 1 |
| USA300 ISMMS1 | CP007176 |  |  | 1 |
| ECT-R 2 | FR714927 |  |  | 1 |

**Table S3**- List of publicly available genomes of reference strains of non-*S. aureus* staphylococci analysed in this study, with sequence accession number*.*

| **Strain** | **Accession No.** |
| --- | --- |
| *Staphylococcus carnosus* subsp. *carnosus* TM300 | AM295250 |
| *Staphylococcus epidermidis* ATCC 12228 | AE015929 |
| *Staphylococcus epidermidis* RP62A | CP000029 |
| *Staphylococcus haemolyticus* JCSC1435 | AP006716 |
| *Staphylococcus lugdunensis* HKU09-01 | CP001837 |
| *Staphylococcus pasteuri* SP1 | CP004014 |
| *Staphylococcus pseudintermedius* ED99 | CP002478 |
| *Staphylococcus pseudintermedius* HKU10-03 | CP002439 |
| *Staphylococcus saprophyticus* subsp. *saprophyticus* ATCC 15305 | AP008934 |
| *Staphylococcus warneri* SG1 | CP003668 |

**Table S4** *–* List of primer sequences used during *in silico* PCR analysis, to identify each gene in the *ess* locus. All primers were generated from the 30 terminal nucleotides of each gene identified from the *ess* loci of four published reference genomes: Newman [28] S0385 [47], MRSA252 [45] and HO 5096 0412 [46]*.*

| **Gene** | **Reference genome from which primers created** | **Forward primer sequence** | **Reverse primer sequence** |
| --- | --- | --- | --- |
| *esxA* | Newman | ATGGCAATGATTAAGATGAGTCCAGAGGAA | ttattgcaaaccgaaattattagaaagttg |
| *esaA* | Newman | ATGAAAAAGAAAAATTGGATTTATGCATTA | cattagattaatctctctttcttaaagtgt |
| *essA* | Newman | ATGTTGATGAATAGCGTGATTGCTTTAACT | tcaaatgttacttttacgtgctgat |
| *esaB* | MRSA252 | ATGAATCAGCACGTAAAAGTAACATTTGAT | ctatagtaacttcaaaatatctccatcagc |
| *essB* | Newman | ATGGTTAAAAATCATAACCCTAAAAATGAA | ctatttttttctttcagcttcttggcgttt |
| *essC1* | Newman | ATGCATAAATTGATTATAAAATATAACAAA | ctatttaaaccatctaatcttttgataagc |
| *essC2* | S0385 | TATAGGACTGAGGCAAAGACAATGCATAAA | ctattgaattaattttattttaatatgttc |
| *essC3* | MRSA252 | ATGCATAAATTGATTATAAAATATAACAAA | ctatccctccattagttttattttttgat |
| *essC4* | HO 5096 0412 | TATAGGACAGAGGCAAAGACAATGCATAAA | ttattcaaataatttaatttttttatattc |
| *esxC* | Newman | ATGAATTTTAATGATATTGAAACAATGGTC | ttaattcattgctttattaaaatattcactt |
| *esxB* | Newman | ATGGGTGGATATAAAGGTATTAAAGCAGAT | tcatgggttcaccctatcaagcccttgctt |
| *esaE* | Newman | ATGAAAGATGTTAAGCGAATAGATTATTTT | ttactcctctgctttattaatatgattttc |
| *esxD* | Newman | ATGACGTTGAGTGGAAAAATTAGTGTTAAA | ctatccctcaatattatagtaaagcttggc |
| *essD** | Newman | GTGCATGACATGACAAAAGATATTGAATAT | ctacataaacttcttttatgcgacctttat |
| *NWMN_0230* | Newman | ATGACTACTAAAGAAAATATTGATACTCTT | ttagtcatcatcttcagtgtttaattcaaa |
| *NWMN_0231* | Newman | ATGGAGTTCTTATTATTAATTGTCGTAGCC | ttataataatcttgataaaatcatattcaa |
| *NWMN_0232* | Newman | ATGGAAAACCAAAAACAAGGCAATGGCTTA | ttatcttaaacctaaatatgctaataaatt |
| *NWMN_0237* | Newman | ATGACTACTAAGGAAAATATAGACATTCTT | ttagtcatcatcttcagtgtttaattcaaa |
| *NWMN_0245* | Newman | ATGAAAAGAATATTGGTAGTATTTTTAATG | ttaatctttttccacttctacattttcatc |
| *NWMN_0246* | Newman | ATGGAAAAATCGATCAAAATAATGACAATA | ttatgataagtcgcttatttcatctttagg |
| *NWMN_0216* | Newman | ATGGAATTGAAAGTCGATGATTTTGTAAAG | ttacattgtcttaccttcattttcgatatc |
| *NWMN_0217* | Newman | ATGATAATTTTTATTTTAATAACAATATTT | ttacgaaagtataattgtagctataataat |
| *SAR0285* | MRSA252 | TTGATAAAGTTAAACCAAGCCTCTGTCAGC | ttatttattaatttgtcctgctatttctcta |
| *SAR0286* | MRSA252 | ATGAGTAATAAGGGAGAAATAAGAAGGCAA | ttataattttgatttaagtgcatctatagc |
| *SAR0287* | MRSA252 | ATGGGGTACAAAGTTGATATGTCTGAAGTG | ttatccgaaaactgttttaaatcctgaagt |
| *SAR0288* | MRSA252 | ATGAAAATACAAGAGATTGTAGGAAATATA | ttaatcatccactccgttttcgatatcttt |
| *SAR0289* | MRSA252 | ATGGAAGTAGGAATGTTAATTATGCCGTTT | ttatccatatacttcactctcatccaaatc |
| *SAR0290* | MRSA252 | ATGATGAAAAATGTAGCTTACAAAACTTAT | ttaatcattttcatacactttggcctttac |
| *SAR0291* | MRSA252 | ATGATAATTTTTATTTTATTAACAGTTTTT | ttatgaaagtacaatagtagctataataat |
| *SAR0292* | MRSA252 | ATGGAAAACCAAAAACAAGGCAATGGCTTA | ttatcttaaacctaaatatgctaataaatt |
| *SAEMRSA15_02470* | HO 5096 0412 | AAGGAGAGTATTATGAGATTTGATTTTAAT | ttaaacctcctcaaagttaattttaaaatt |
| *SAEMRSA15_02480* | HO 5096 0412 | ATGGGGAAAGAAATTTATTTAGATCCAATT | ctaattcctatttgcaattttatttgcatt |
| *SAEMRSA15_02490* | HO 5096 0412 | ATGCCTAGATTAGTTAATTCAGAGTTAGAA | ttaccatgtaattttaatttttcctccaag |
| *SAEMRSA15_02500* | HO 5096 0412 | ATGTCGAAATCATCTTTAATATTGGAAAAG | ttaattagttaatttttcaatagcagcagc |
| *SAEMRSA15_02510* | HO 5096 0412 | ATGGAAAAAATAAAGAGAATAAACAAAATA | ttatctatttttaataaaaaagtctttcaa |
| *SAEMRSA15_02520* | HO 5096 0412 | ATGAAAAGATTAATTATAGGAGTGTTAATT | ttattttactttaatatctttataatctga |
| *SAEMRSA15_02530* | HO 5096 0412 | ATGGAAAAATTAATCAAAGTGACGTCTTTA | ctatgacacttcttttcctattttgttata |
| *SAEMRSA15_02540* | HO 5096 0412 | ATGAAATATACTTGGTGGATACTTTTAACA | ctattgctttaatcttgattgctgtatttt |
| *SAPIG0303* | S0385 | ATGGGGGAAATAAAAGTTGAAACGAGTAGT | ttataatcctgctcctaaattattatttcc |
| *SAPIG0304* | S0385 | TTGAAAAGCTTACAAGGAGATAAGAAAAGG | ttataattgactgtctatattttgaatttc |
| *SAPIG0305* | S0385 | ATGGGGAATAAAATAAAAATGTCAGAAGTG | ttaaaatacattgcttaacgttttaccaaa |
| *SAPIG0306* | S0385 | ATGAATAATACTAAGGAAAATATTGATACT | ttagtcatcatctgctgtgtttaattcaaa |
| *SAPIG0307* | S0385 | ATGGAACTTGATGCATTAGTAATGCCTAAC | tcattccaattcatcctcatctaaattgat |
| *SAPIG0308* | S0385 | ATGAATGAATTAAGCTATTCTACTATAATG | tcaatattccataacttttactttaatatc |
| *NWMN_0559* | Newman | ATGAGTATTGACATGTATTTAGACAGATCT | ttcgatgaaatggcttgctgattatcggaa |

*Due to variation at the 3’ end of the *essD* and *NWMN_0559* genes, a reverse primer was developed within the conserved sequence upstream of this variable region, to determine the presence these genes in each isolate.

a)

**Table S5**: RNA-Seq transcriptional analysis of the *ess* clusters. FPKM values for each sample separately and averaged across the three samples for the strains:

a) S0835 b) MRSA252 c) HO 5096 0412 d) NCTC 8325

|  | GeneID | FPKM Sample 1 | FPKM Sample 2 | FPKM Sample 3 | Averaged FPKM |
| --- | --- | --- | --- | --- | --- |
| Module 1 | esxA | 33127 | 31624 | 35687 | 33479 |
|  | esaA | 532.83 | 492.80 | 522.99 | 516.20 |
|  | essA | 619.45 | 566.51 | 611.27 | 599.08 |
|  | esaB | 205.12 | 121.98 | 144.76 | 157.29 |
|  | essB | 379.11 | 316.05 | 361.31 | 352.16 |
| Module 2 | essC | 294.45 | 236.36 | 268.34 | 266.38 |
|  | SAPIG0303 | 179.77 | 124.44 | 160.47 | 154.89 |
|  | SAPIG0304 | 236.94 | 167.96 | 213.42 | 206.11 |
|  | SAPIG0305 | 379.63 | 359.81 | 392.23 | 377.22 |
|  | SAPIG0306 | 278.34 | 276.60 | 292.47 | 282.47 |
|  | SAPIG0307 | 404.18 | 407.32 | 434.86 | 415.45 |
|  | SAPIG0308 | 62.568 | 59.607 | 72.919 | 65.031 |
| Module 3 | SAPIG0309 | 804.75 | 896.32 | 927.16 | 876.08 |
|  | SAPIG0310 | 233.08 | 210.54 | 205.37 | 216.33 |
|  | SAPIG0311 | 344.46 | 332.01 | 331.83 | 336.10 |
|  | SAPIG0312 | 27.412 | 24.822 | 24.265 | 25.499 |
|  | SAPIG0313 | 893.57 | 751.28 | 877.75 | 840.87 |
|  | SAPIG0314 | 187.22 | 179.42 | 148.69 | 171.78 |
|  | SAPIG0315 | 174.89 | 133.46 | 127.27 | 145.20 |
| Module 4 | SAPIG0316 | 475.21 | 411.73 | 430.65 | 439.19 |
|  | SAPIG0317 | 2333.5 | 1834.5 | 2123.5 | 2097.2 |

b)

|  | GeneID | FPKM Sample 1 | FPKM Sample 2 | Averaged FPKM |
| --- | --- | --- | --- | --- |
| Module 1 | esxA | 2198.3 | 2263.3 | 2230.84 |
|  | esaA | 27.674 | 31.411 | 29.542 |
|  | essA | 26.776 | 32.982 | 29.879 |
|  | esaB | 17.739 | 15.499 | 16.619 |
|  | essB | 16.995 | 19.660 | 18.328 |
| Module 2 | essC | 16.542 | 17.664 | 17.103 |
|  | SAR0285 | 4.9066 | 4.9536 | 4.9301 |
|  | SAR0286 | 10.893 | 10.453 | 10.673 |
|  | SAR0287 | 29.145 | 29.514 | 29.330 |
|  | SAR0288 | 52.823 | 55.482 | 54.152 |
|  | SAR0289 | 108.46 | 115.42 | 111.94 |
|  | SAR0290 | 116.08 | 145.79 | 130.94 |
| Module 3 | SAR0291 | 130.49 | 147.47 | 138.98 |
|  | SAR0292 | 345.47 | 473.37 | 409.42 |
|  | SAR0293 | 2.6554 | 3.0571 | 2.8563 |
|  | SAR0294 | 3.0642 | 2.9474 | 3.0058 |
|  | SAR0295 | 1.6686 | 2.2509 | 1.9597 |
|  | SAR0297 | 2.4271 | 2.6921 | 2.5596 |
| Module 4 | SAR0299 | 19.464 | 23.415 | 21.440 |
|  | SAR0301 | 126.69 | 161.20 | 143.94 |

c)

|  | GeneID | FPKM Sample 1 | FPKM Sample 2 | FPKM Sample 3 | Averaged FPKM |
| --- | --- | --- | --- | --- | --- |
| Module 1 | esxA | 9428.5 | 9517.7 | 10740 | 9895.5 |
|  | esaA | 123.31 | 148.53 | 154.98 | 142.27 |
|  | essA | 121.33 | 147.71 | 159.98 | 142.90 |
|  | esaB | 68.558 | 68.918 | 97.151 | 78.209 |
|  | essB | 75.467 | 85.013 | 80.454 | 80.311 |
| Module 2 | essC | 59.886 | 68.081 | 71.168 | 66.378 |
|  | SAEMRSA15_02470 | 31.044 | 34.705 | 31.0922 | 32.280 |
|  | SAEMRSA15_02480 | 42.762 | 39.638 | 42.8485 | 41.750 |
|  | SAEMRSA15_02490 | 35.584 | 31.832 | 38.4615 | 35.292 |
|  | SAEMRSA15_02500 | 224.31 | 202.17 | 204.694 | 210.39 |
| Module 3 | SAEMRSA15_02510 | 116.33 | 137.51 | 130.925 | 128.26 |
|  | SAEMRSA15_02520 | 130.55 | 138.70 | 139.549 | 136.27 |
|  | SAEMRSA15_02530 | 508.68 | 559.25 | 541.154 | 536.36 |
|  | SAEMRSA15_02540 | 365.20 | 400.53 | 397.978 | 387.90 |
|  | SAEMRSA15_02550 | 0 | 0 | 0 | 0 |
|  | SAEMRSA15_02560 | 0 | 0 | 0 | 0 |
|  | SAEMRSA15_02570 | 6.1642 | 4.8318 | 7.01293 | 6.0030 |
|  | SAEMRSA15_02580 | 7.6279 | 5.4155 | 7.89885 | 6.9807 |
| Module 4 | SAEMRSA15_02590 | 153.05 | 150.15 | 151.543 | 151.58 |
|  | SAEMRSA15_02600 | 340.64 | 337.26 | 444.306 | 374.07 |

|  | GeneID | FPKM Sample 1 | FPKM Sample 2 | FPKM Sample 3 | Averaged FPKM |
| --- | --- | --- | --- | --- | --- |
| Module 1 | esxA | 13714 | 13684 | 14140 | 13846 |
|  | esaA | 310.46 | 310.98 | 291.71 | 304.39 |
|  | essA | 352.41 | 349.08 | 322.35 | 341.28 |
|  | esaB | 92.123 | 107.52 | 106.07 | 101.90 |
|  | essB | 148.33 | 162.97 | 155.11 | 155.47 |
| Module 2 | essC | 140.65 | 149.78 | 147.79 | 146.07 |
|  | esxC | 205.80 | 126.68 | 167.90 | 166.79 |
|  | esxB | 228.84 | 195.27 | 220.34 | 214.82 |
|  | esaE | 114.40 | 98.552 | 97.413 | 103.45 |
|  | esxD | 205.10 | 152.05 | 196.97 | 184.71 |
|  | essD | 135.66 | 127.11 | 128.06 | 130.28 |
| Module 3 | SAOUHSC_00269 | 953.95 | 945.82 | 856.26 | 918.68 |
|  | SAOUHSC_00270 | 123.61 | 119.02 | 122.45 | 121.70 |
|  | SAOUHSC_00271 | 83.945 | 71.821 | 68.677 | 74.814 |
|  | SAOUHSC_00272 | 448.11 | 417.42 | 348.61 | 404.71 |
|  | SAOUHSC_00274 | 22.963 | 25.333 | 19.160 | 22.486 |
|  | SAOUHSC_00275 | 24.425 | 23.863 | 16.893 | 21.727 |
|  | SAOUHSC_00276 | 32.239 | 33.054 | 23.021 | 29.438 |
|  | SAOUHSC_00277 | 36.505 | 32.891 | 25.630 | 31.675 |
|  | SAOUHSC_00278 | 24.332 | 21.769 | 22.646 | 22.916 |
| Module 4 | SAOUHSC_00279 | 741.34 | 727.21 | 651.54 | 706.70 |

d)
